# Development of a Novel Wasp-Inspired Friction-Based Tissue Transportation Device

**DOI:** 10.3389/fbioe.2020.575007

**Published:** 2020-09-30

**Authors:** Aimée Sakes, Ivo A. van de Steeg, Esther P. de Kater, Perry Posthoorn, Marta Scali, Paul Breedveld

**Affiliations:** Bio-Inspired Technology Group, Faculty of Mechanical, Maritime, and Materials Engineering, Department of BioMechanical Engineering, Delft University of Technology, Delft, Netherlands

**Keywords:** bio-inspired design, biomimetics, medical device design, minimally invasive surgery, ovipositor, surface-dependent friction, tissue transportation

## Abstract

Currently existing tubular transportation systems for the extraction of large tissue masses during Minimal Invasive Surgery (MIS) are subjected to a large amount of operating limitations. In this study, a novel transportation mechanism (patented) was developed inspired by the egg-laying structure of wasps. The developed mechanism consists of an outer tube within which six reciprocating semi-cylindrical blades are present and tissue is transported using a friction differential between the blades. Two motion sequences were developed: (1) 1–5 motion sequence, in which one blade moves forward, while the remaining five blades move backward and (2) 2–4 motion sequence, in which four blades move backward while two blades move forward. A proof-of-principle experiment was performed to investigate the effects of tissue elasticity, tissue heterogeneity, and the motion sequence on the transportation rate [mg/s], transportation efficiency [%], and transportation reliability [%]. The mean transportation rate and reliability was highest for the 9 wt% gelatine phantoms at 4.21 ± 0.74 mg/s and the 1–5 sequence at 100%, respectively. The prototype has shown that the friction-based transportation principle has the potential of becoming a viable and reliable alternative to aspiration as a transportation method within MIS.

## Introduction

### Tissue Extraction

The extraction of tissues from the human body is an important procedure found in many fields of surgery. Common reasons for tissue extraction are the examination of suspicious lesions inside the body (Parker et al., 1995) and the removal of tumorous (Koffron et al., 2007; Buell et al., 2008), dying (Steed, 2004), and infected tissues (Steed, 2004) from the patient during open or Minimally Invasive Surgery (MIS).

While the extraction of superficial tissues is generally a straight-forward procedure, extraction of tissues deeper into the body is more challenging. In an effort to facilitate tissue extraction at remote locations in the human body, a number of transportation mechanisms have been proposed that allow for tissue extraction during the procedure, including aspiration-based devices, such as aspiration catheters (Madjidyar et al., 2019), and flexible graspers, such as bioptomes (Fowles and Mason, 1984) and stent-retrievers (Spiotta et al., 2015). The current “golden standard” in tissue transportation are aspiration-based devices that use a pressure differential for tissue transportation in order to facilitate fast extraction and prevent debris from pilling up at the operation site.

### Current Challenges in Tissue Transportation Systems

In the on-going drive for miniaturisation and MIS at remote locations in the body, aspiration-based transportation falls short due to the inability to create a sufficiently high pressure-differential in long and narrow tubes. For example, in a study of Madjidyar et al. (2019), it was found that the efficacy of thrombectomy using an aspiration-catheter depends on thrombus composition. In their study, they were unable to aspirate fibrin-rich clots using the ADAPT catheter (SOFIA 5F, Microvention, United States). Aspiration of “softer” erythrocyte-rich clots was also unsuccessful in two out of the six attempts. Even though the authors do not offer an explanation as to why the clots could not be aspirated, we hypothesize that the combination of insufficient pressure differential, large size of the clots, and the clot composition are the main contributors. As the field of MIS progresses, further downsizing of the diameter of tissue transportation systems is a necessity, making aspiration less of an option in future. Additionally, aspiration-based transportation devices are prone to clogging and tissue lumps that are aspirated into a tube may get damaged, which can negatively affect subsequent investigation in the case of a biopsy. Furthermore, the aspiration-force not only affects the harvested tissue, but also the surrounding tissues, which might result in damage to healthy tissues when they accidentally get pulled into the tube and, in an environment containing several types of liquid and tissues, indiscriminate tissue transport.

### Goal of This Study

As of today, there are no good alternatives for aspiration-based tissue transport. The inability to reliably transport tissues is a recurring problem in cardiology (Pornratanarangsi et al., 2008; Madjidyar et al., 2019), gynaecology (Cohen and Greenberg, 2011), and neurology (Yoo and Andersson, 2017) (amongst others). The goal of this study is, therefore, to develop a new method of tissue transport for MIS, which allows for reliable continuous transportation of tissue through long and narrow tubes and does not affect the surrounding tissues.

## Proposed Transportation System

### Bio-Inspiration: Wasp-Ovipositor Egg Transport

**FIGURE 1:**
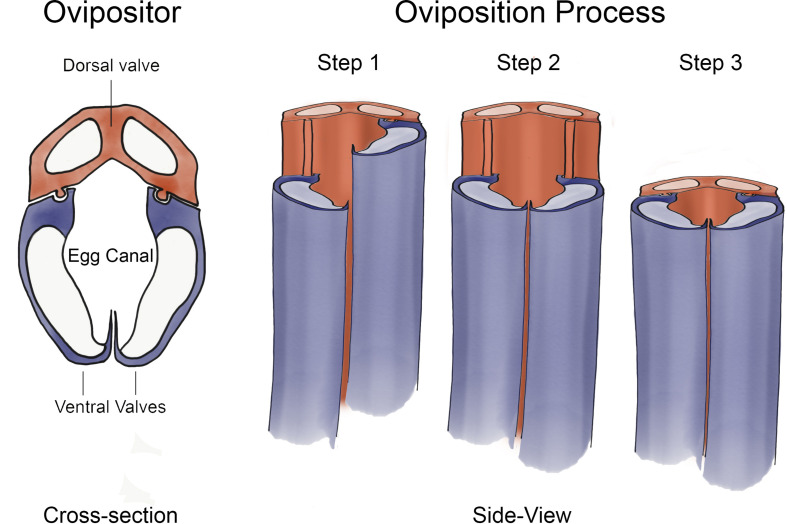
Schematic Representation of the Working Principle of the Oviposition Process. The substrate is penetrated by moving the valves in an alternating fashion. (1) One of the ventral valves moves deeper into the substrates using the dorsal valve as sliding support. (2) The second ventral valve moves deeper into the substrate, also using the dorsal valve as sliding support. (3) The dorsal valve penetrates the substrate using both ventral valves as sliding support.

Our new transportation system is based on the egg-laying needle, also known as the ovipositor, of some species of parasitic wasps (Vincent and King, 1995; Le Lannic and Nénon, 1999; Kundanati and Gundiah, 2014; Nakajima and Schwarz, 2014). The purpose of the ovipositor is the deposition of eggs in a host, hidden in a piece of fruit (Kundanati and Gundiah, 2014), or the bark of a tree (Le Lannic and Nénon, 1999) in order to provide the wasp’s progeny with nutrition during the early stages of development (Vincent and King, 1995; Nakajima and Schwarz, 2014). The ovipositor generally consists of three segments called valves (Figure 1) (Quicke et al., 1994; Rahman et al., 1998). The largest segment is called the dorsal valve and is connected by means of a tongue-and-groove mechanism, called olistheter, to the other two valves, called the ventral valves. The olistheter consists of a tongue (rachis) on the dorsal valve and a groove (aulax) on the ventral valve and allows the valves to slide relative to each other while staying parallel (Quicke et al., 1994; Rahman et al., 1998).

The exact working principle of the oviposition process is still under investigation. However, in a study of Cerkvenik et al. (2017), two main methods of ovipositor insertion were identified: (1) pushing of the entire ovipositor with minimal relative valve movement and (2) inserting the ovipositor with alternating valve movement of low amplitude, referred to as the “surface-dependent friction” method. According to the “surface-dependent friction” method, the wasp penetrates the substrate by moving the valves of the ovipositor in an alternating fashion (Vincent and King, 1995). In Figure 1, a stylized version of an ovipositor is illustrated. The oviposition process using alternating valve movement can be characterized by three consecutive steps. First, one of the ventral valves moves deeper into the substrate using the dorsal valve as sliding support. Subsequently, the second ventral valve moves deeper into the substrate, also using the dorsal valve as sliding support. Thirdly, the dorsal valve moves deeper into the substrate using both the ventral valves as sliding support. By only moving one valve at the time, it is hypothesized that the friction of the remaining two valves with the substrate is higher than the friction of the one valve with the substrate due to the difference in surface area, allowing for easy penetration of the substrate with minimal net push force to overcome the penetration force of the substrate. It is hypothesized that the motion of the valves is also used to move the egg along the inside of the ovipositor (Austin and Browning, 1981).

### Bio-Inspired: Wasp-Ovipositor Tissue Transportation

#### Surface-Dependent Friction Transportation: Working Principle

Inspired by the surface-dependent friction method hypothesized for ovipositor insertion in parasitic wasps, at Delft University of Technology a series of self-propelling needles have been developed (Sprang et al., 2016; Scali et al., 2017, 2019). The needles consist out of four to six equally sized rods that can be individually controlled and moved in the axial direction. Experiments with these needles have shown that the surface-dependent friction method can be used to propel the needle through gelatine tissue phantoms using minimal net push force (Sprang et al., 2016) and even steer (Scali et al., 2017, 2019).

**FIGURE 2:**
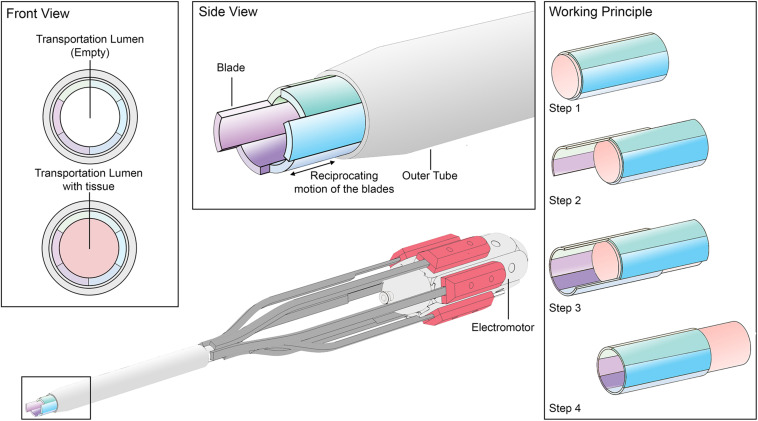
Schematic representation of the developed surface-dependent friction transportation system and its working principle.

Based on the results of these studies, we decided to turn the system inside out; instead of using the surface-dependent friction method for propelling the needle through a medium, we use the surface-dependent friction method to propel a medium through the system. The developed transportation system consists of six blades held together by an outer tube (Figure 2). The semi-cylindrical blades are equal in size and shape. Together the blades enclose the tissue transportation lumen. In order to initiate tissue transport, the blades are actuated using an electromotor. Each blade can translate in the axial direction independently from its neighbouring blades. Once a piece of tissue with an equal diameter as the transportation lumen enters the transportation system, tissue transportation will commence by axially translating the blades in a consecutive fashion. In order to initiate transport, the tissue that needs to be transported through the transportation lumen needs to undergo a resultant friction force with the blades in the desired transportation direction. This resultant friction force is comprised of six frictional components; one for each blade with the tissue (Eq. 1). If we assume that gravity can be neglected and the tissue has full contact with all six blades during transportation, it can be stated that each blade contributes one sixth of the total friction with the tissue (Eq. 2). Thus, if one blade is retracted and the remaining five blades are kept stationary, the friction between the stationary blades and the tissue is five times as large (5/6 of the total friction) as that of the retracting blade and the tissue (1/6 of the total friction). If the majority of the blades is retracted, the friction between the majority of the blades with the tissue (>3/6 of the total friction) exceeds the friction between the minority of the blades with the tissue (<3/6 of the total friction), which results in tissue transportation in the direction of the majority of the blades (Eqs 3 and 4). By systematically changing the number of blades that retreat, are stationary, and advance per actuation cycle, tissue transportation can be achieved in both directions using only a limited stroke length of the blades.

(1) $$\begin{array}{c} {\vec{F}}_{f\,r\,i\,c\,t\,i\,o\,n}={\vec{F}}_{f\,r\,i\,c\,t\,i\,o\,n\,1}+{\vec{F}}_{f\,r\,i\,c\,t\,i\,o\,n\,2}+{\vec{F}}_{f\,r\,i\,c\,t\,i\,o\,n\,3}+{\vec{F}}_{f\,r\,i\,c\,t\,i\,o\,n\,4}\\ +{\vec{F}}_{f\,r\,i\,c\,t\,i\,o\,n\,5}+{\vec{F}}_{f\,r\,i\,c\,t\,i\,o\,n\,6} \end{array}$$

(2) $$\begin{array}{c} \left|{\vec{F}}_{f\,r\,i\,c\,t\,i\,o\,n\,1}\right|=\left|{\vec{F}}_{f\,r\,i\,c\,t\,i\,o\,n\,2}\right|=\left|{\vec{F}}_{f\,r\,i\,c\,t\,i\,o\,n\,3}\right|=\left|{\vec{F}}_{f\,r\,i\,c\,t\,i\,o\,n\,4}\right|\\ =\left|{\vec{F}}_{f\,r\,i\,c\,t\,i\,o\,n\,5}\right|=\left|{\vec{F}}_{f\,r\,i\,c\,t\,i\,o\,n\,6}\right|=\frac{1}{6}\,{\vec{F}}_{f\,r\,i\,c\,t\,i\,o\,n\,6} \end{array}$$

(3) $$\sum {\vec{F}}_{f\,r\,i\,c\,t\,i\,o\,n\,a\,d\,v\,a\,n\,c\,i\,n\,g\,b\,l\,a\,d\,e\,s}\neq \sum {\vec{F}}_{f\,r\,i\,c\,t\,i\,o\,n\,r\,e\,t\,r\,e\,a\,t\,i\,n\,g\,b\,l\,a\,d\,e\,s}$$

(4) $$\sum {\vec{F}}_{f\,r\,i\,c\,t\,i\,o\,n\,a\,d\,v\,a\,n\,c\,i\,n\,g\,b\,l\,a\,d\,e\,s}\vee \sum {\vec{F}}_{f\,r\,i\,c\,t\,i\,o\,n\,r\,e\,t\,r\,e\,a\,t\,i\,n\,g\,b\,l\,a\,d\,e\,s}>\frac{3}{6}\,{\vec{F}}_{f\,r\,i\,c\,t\,i\,o\,n}$$

with:

${\vec{F}}_{\text{friction}}$ = resultant friction force [N] of the blades with the tissue.

${\vec{F}}_{\mathrm{friction1},2,3,4,5,6}$ = friction force [N] with the tissue of blade 1–6, respectively.

When taking the effect of gravity into account, Eq. 2 will no longer hold. Instead, the friction force per blade will depend on the position of this blade in the instrument’s shaft and the instrument’s orientation. The friction force is the result of the perpendicular component of the gravitational force exerted on the blade, the normal force exerted by the elastically compressed tissue on the blade, the friction coefficient between the blade and the tissue, and the surface area between the tissue and the blade. Since the size of the perpendicular component of the gravitational force is dependent on the instrument’s and blade’s orientation, unequal frictional forces between the blades and the tissue can occur. This can potentially negatively affect tissue transportation if the difference in friction between the retreating blades and the tissue and advancing blades and the tissue becomes close to zero. This does not hold, for a vertical instrument orientation. In this case, the friction force between each blade and the tissue is equal, since the gravitational force does not generate any normal force. However, this would require a very high friction coefficient between the blades and the tissue to fully compensate the sliding force generated by the weight of the tissue. Due to the small scale of the device, the mass of the transported tissue is very small, as a result of which the effect of gravity on tissue transportation is minimal. Gravity is, therefore, neglected in this paper.

#### Surface-Dependent Friction Transport: Proof-of-Principle Prototype

**FIGURE 3:**
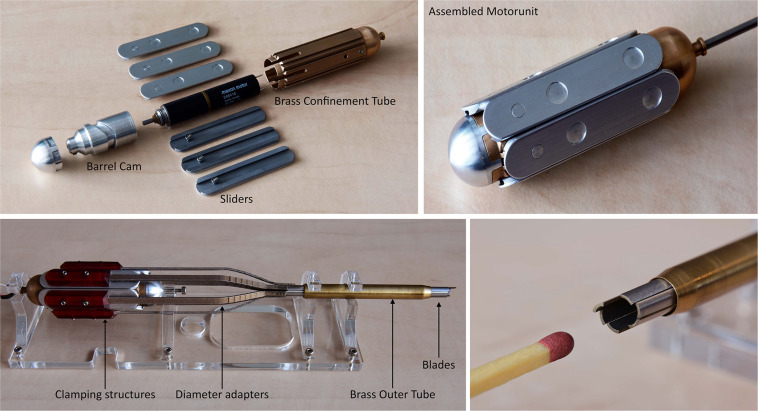
Assembled prototype.

Figure 3 shows the developed prototype. The blades are actuated using an electromotor (248416 Maxon DC Motor fitted with 256:1 gear box, Sachseln, Switzerland) connected to an aluminium barrel cam surrounded by a V-shaped slot. The barrel cam converts the rotational motion of the electromotor to an axial translating motion of the blades. In the curved slot of the barrel cam, six miniature ball bearings are placed that are connected to aluminium sliders. The electromotor and barrel cam are confined within a brass cylindrical frame. The brass cylindrical frame fixes the electromotor in place and provides a smooth, low frictional, gliding surface for the sliders. Per slider, a 3D-printed (R5, Perfactory Standard, EnvisionTec, Gladbeck, Germany) clamping structure was attached, that is in turn connected to a curved and slender diameter adapter that was 3D-printed (Mysint 100 PM, Sisma, Schio, Italy) out of stainless steel in order to maintain a balance between a thin design and a sufficiently stiff construction. Finally, these adapters were attached to the blades at the distal end using glue. Two sets of six blades were manufactured by means of Electrical Discharge Machining (EDM) out of a stainless-steel capillary tube (Ø_outer_ 5 mm, wall-thickness *t* = 0.5 mm, *L* = 80 mm). The six blades were placed within a tapered brass tube (Ø_outer_ 7 mm, wall-thickness *t* = 1 mm, *L* = 60 mm) to prevent outward motion of the blades. The prototype is suspended in a frame that was lasercut from 5 mm thick transparent PolyMethylMethaAcrylate (PMMA). In the Supplementary Material, a video illustrating the working principle and tissue transportation capability of the developed wasp-inspired transportation device can be found.

## Proof-of-Principle Experiment

### Experiment Goal

In a proof-of-principle experiment, the functioning of the developed surface-dependent friction transportation prototype was evaluated. The main goal of the experiment was threefold: (1) determine the feasibility of using the surface-dependent friction principle for transportation of tissues with varying elasticity and heterogeneity, (2) determine the effect of the motion sequence of the blades on the efficiency and reliability of the tissue transportation, and (3) evaluate the performance of the prototype in terms of the achieved transportation rate [mg/s] and achieved transportation efficiency [%]. The transportation prototype was not tested on its effects on the surrounding tissues. The developed prototype is inherently safe to surrounding tissues by design, as it does not incorporate aspiration as a means of tissue transportation, the blades are shielded by the brass outer tube, and the distal end of the prototype is blunt; preventing unwanted damage to surrounding tissues.

### Experiment Variables

#### Independent Variables

##### Tissue elasticity

In order to determine the feasibility of using the surface-dependent friction principle to transport different types of tissues, three gelatine tissue phantoms were manufactured. Gelatine has been shown to mimic mechanical characteristics of skin and organ tissues (Misra et al., 2009; Cournane et al., 2012; Farrer et al., 2015) and is, therefore, a suitable tissue phantom to determine the feasibility of using surface-dependent friction for tissue transportation. Soft tissues and organs have a Young’s modulus that ranges from 0.1 kPa for brain tissue to approximately 800 kPa for cartilage (Arda et al., 2011; Hoenig et al., 2013). In order to evaluate the mechanical performance of the prototype for different tissue elasticity, three different gelatine tissue phantoms were manufactured out of 6, 9, and 12 weight percentage (wt%) gelatine mixed with tap water, which represent a compressive Young’s modulus range of approximately 27–74 kPa (Karimi and Navidbakhsh, 2014). This Young’s modulus range represent a large range of tissues and organs, such as the renal cortex and pelvis, glandular tissues, muscles, and tendons, that can be encountered during MIS.

##### Tissue heterogeneity

Organs and tissues are often heterogeneous in nature. Especially tumorous tissues, which preferably need to be removed and transported out of the body of the patient to prevent further harm, have been found to be heterogeneous in structure (Polyak, 2011; Chapman et al., 2014; Heindl et al., 2015). In order to test the assumption that the developed prototype is able to transport heterogeneous tissues, the gelatine tissue phantoms were mixed with Ø2 mm rigid granules (0.25 g) to create heterogeneity.

##### Motion sequence

In the proposed surface-dependent friction principle, tissue transportation is achieved by using the difference in friction between the retreating and advancing blades. In order to determine the effect of the number of retreating and advancing blades, two motion sequences; the 1–5 and 2–4 motion sequence, were developed and tested on their effiency and reliability of tissue transport.

###### 1–5 motion sequence (Figure 4, left)

In this motion sequence, one blade advances, while the remaining five blades are retracted in a consecutive fashion. In this motion sequence, the friction exerted upon the tissue by the retreating blades is approximately five times higher than the friction exerted on the tissue by the advancing blade, resulting in tissue transportation in the direction of the retreating blades. The sequence rotates one blade counter clockwise per actuation cycle with a stroke length of 5.2 mm.

**FIGURE 4:**
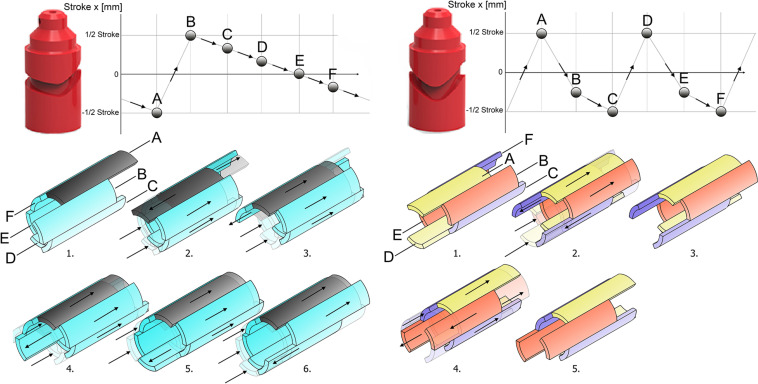
Graphic representation of the 1–5 **(left)** and 2–4 **(right)** reciprocating motion sequence. **(Left)** 1–5 Motion Sequence with associated cam design. In this motion sequence, one blade is advanced, while the remaining blades are gradually retreated in a consecutive manner (1–6). The advancing blade moves one blade counter clockwise per actuation cycle (1–6). **(Right)** (2–4) Motion Sequence with associated cam design. In this motion sequence the blades are divided into pairs: yellow, orange, and purple. First (1–2), the yellow and orange blade pair are retreated, while the blue pair is advanced. Second (3–4), the yellow and blue blade pair are retreated, while the orange blade pair is advanced. Third (5), the sequence rotates one blade pair clockwise and starts by retreating the orange and purple blade pair.

###### 2–4 motion sequence (Figure 4, right)

The blades of this motion sequence are divided into three pairs: the yellow pair, the orange pair, and the blue pair; effectively transforming the six blades into three blades, each with double surface area. In this configuration, two blade pairs retreat, while one blade pair advances. The friction exerted upon the tissue by the retreating pairs is approximately twice as large as the friction exerted on the tissue by the advancing pair. In an effort to reduce the effect of the orientation of the prototype’s shaft, the blades of each pair oppose each other to minimize the effect of gravity, as well as minimize a possible variation in the amount of surface contact between the pairs when transporting irregularly-shaped pieces of transportable tissue. The sequence rotates one blade pair clockwise per actuation cycle with a stroke length of 5.7 mm.

#### Dependent Variables

##### Transportation rate

During MIS, time is critical. The surgery needs to be performed as fast as possible in order to minimize general anaesthesia time and operation cost, while maintaining safety. Therefore, the transportation rate, defined as the amount of time *t* [s] required to transport 1 mg of gelatine tissue phantom through the lumen of the developed prototype, needs to be high to accommodate fast removal of large tissue masses. As the maximum transportation rate of our prototype is mainly dependent on the rotational velocity of the chosen electromotor, we decided to focus on comparing the transportation rate between the gelatine tissue phantoms. The transportation rate per transportation cycle was determined by dividing mass *m* (mg) of the gelatine tissue phantom by the time *t* (s) it took to transport this phantom through the transportation lumen.

##### Transportation efficiency

For both motion sequences, the rotational velocity of the electromotor was kept constant at 46 rpm, resulting in an average stroke velocity of the blades of 4 mm/s and 8.77 mm/s in the 1–5 motion sequence and 2–4 motion sequence, respectively. The achieved transportation velocity is determined by dividing the length of the blades (*L* = 80 mm; transported distance) by the time *t* [s] it takes to transport the tissue over this distance. The achieved transportation velocity will be compared with the theoretical maximum achievable transportation velocity per motion sequence, which will give an indication on the transportation efficiency, and thus tissue slip, during transport.

##### Transportation reliability

The reliability of a medical instrument is of vital importance to allow for a safe and effective MIS procedure. In the proposed transportation system, the ability to transport the tissue from the distal tip to the handle is the most important variable that determines overall reliability. The reliability rate was defined as the total number of times the prototype was able to transport the tissue from the tip toward the handle, subdivided by the total number of tests, multiplied by 100%. The reliability rate was measured for each condition.

### Experimental Facility

**FIGURE 5:**
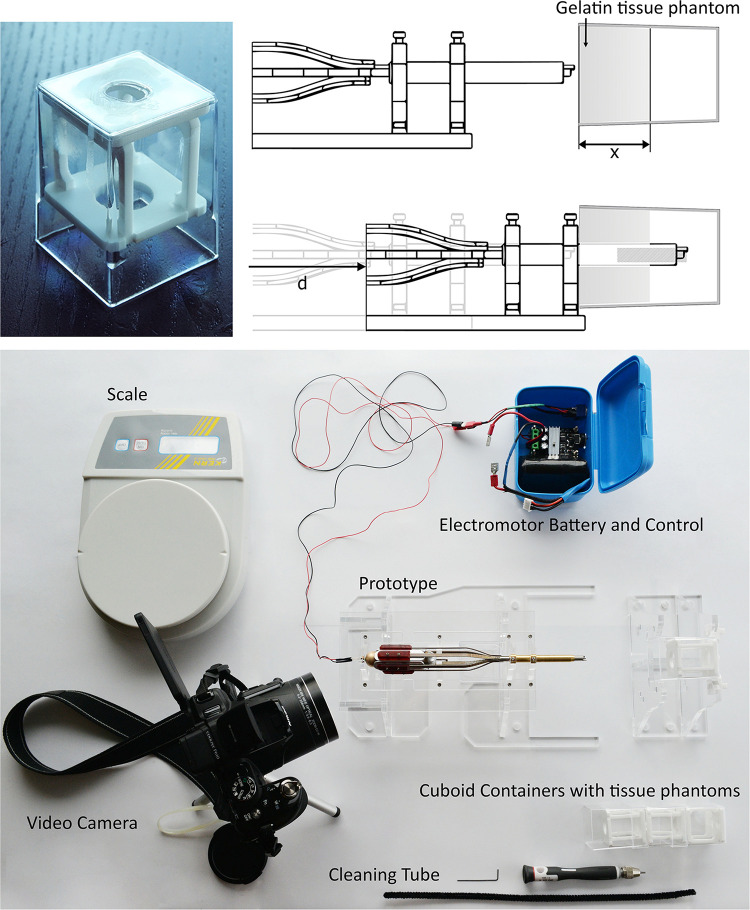
Experimental facility. The variable *d* is defined as the axial translation of the prototype inside the gelatine phantom and is equal to 30 mm. The variable *x* is defined as the thickness of the gelatine phantom and is equal to 20 mm.

The experimental facility consisted of the prototype mounted in the PMMA standard (Figure 5). The gelatine tissue phantoms were contained within tapered 50 ml cuboid containers (35 mm × 38 mm × 52 mm), and were horizontally aligned with the prototype. The cuboid containers all contained a 20 mm thick layer of gelatine and were refrigerated for 14 h before testing. Before the experiment, the proof-of-principle prototype was axially translated over a distance *d* of 30 mm into the cuboid containers to ensure the full 20 mm thick layer of gelatine was contained within the transportation lumen (Figure 5). The electromotor was powered by a 12 V battery, of which the voltage was manually regulated to keep the rotational velocity at 46 rpm. All experiments were recorded with a video camera (Nikon COOLPIX P610).

### Experiment Protocol

The proof-of-principle experiment was subdivided into two sub-experiments.

#### Effect of Tissue Elasticity and Heterogeneity on the Transportation Rate and Reliability

In this experiment, the effect of the gelatine tissue phantoms’ elasticity and heterogeneity on the transportation rate and reliability were determined using the 1–5 motion sequence of the blades.

#### Effect of the Motion Sequence on the Transportation Rate, Transportation Efficiency, and Reliability

In this experiment, the 1–5 and the 2–4 reciprocating motion sequences of the blades were evaluated on their transportation rate, transportation efficiency, and reliability rate using the gelatine phantom that illustrated the highest transportation rate in sub-experiment 1.

Between all experiments and between each repetition, the tube was mechanically cleared of any debris on the inside by pushing a Ø5 mm pipe cleaner several times through the entirety of the tissue transportation lumen. Each condition was tested six times.

### Data Analysis

The time required to transport the gelatine tissue phantom through the transportation lumen was assessed by means of visual analysis of the video recordings. The most successful experimental condition of each sub-experiment was evaluated by determining the highest transportation rate within all data sets of each sub-experiment. The mean transportation rate and the standard deviation, as well as the reliability rate, of each experimental condition were determined per condition. The mean transportation velocity, transportation efficiency, and standard deviation was determined in sub-experiment 2. The statistical analysis was conducted by performing ANOVA analyses and *t*-tests on the data. All data analysis was performed with MATLAB R2015b (The Mathworks, Inc., Natick, MA, United States).

### Results Proof-of-Principle Experiment

#### Tissue Elasticity and Heterogeneity

**FIGURE 6:**
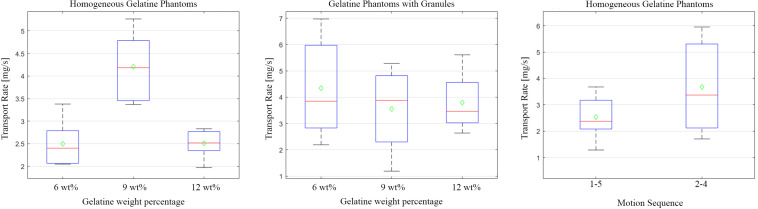
Boxplot graph displaying the transportation rate [mg/s] as the function of the gelatine weight percentage [wt%] and motion sequence. **(Left)** Distribution of data as collected from the homogeneous gelatine tissue phantoms with a weight percentage of: 6, 9, and 12% of sub-experiment I using the 1–5 motion sequence. **(Middle)** Distribution of data as collected from the heterogenous gelatine tissue phantoms with a weight percentage of: 6, 9, and 12% of sub-experiment I using the 1–5 motion sequence. **(Right)** Distribution of data as collected from the 1–5 **(left)** and 2–4 (**right**) motion sequences of sub-experiment II using the homogeneous 9wt% gelatine tissue phantom. The maximum values and minimum values are indicated by the outermost horizontal solid lines on each box plot. The median of each dataset is indicated by the horizontal red line in each boxplot, while the mean is indicated by the green diamond.

In Figure 6, the transportation rate for the homogeneous gelatine tissue phantoms and the gelatine tissue phantoms with granules are graphically displayed using the 1–5 motion sequence. The mean transportation rates for the 6, 9, and 12 wt% homogeneous gelatine tissue phantoms were 2.50 ± 0.54, 4.21 ± 0.74, and 2.51 ± 0.34 mg/s, respectively. There was a statistically significant difference in the transportation rate of the homogeneous gelatine tissue phantoms of different densities as determined by the one-way ANOVA [*F*(2,13) = 16.25, *p* = 0.0003]. The average transportation rates for 6, 9, and 12 wt% gelatine tissue phantoms with granules were 4.34 ± 1.95, 3.56 ± 1.75, and 3.80 ± 1.27 mg/s, respectively. There was no statistically significant difference in transportation rate between the different batches of gelatine tissue phantoms with granules as determined by a one-way ANOVA [*F*(2,10) = 0.25, *p* = 0.78]. Furthermore, there was also no significant difference in transportation rates between the homogeneous gelatine tissue phantoms and the phantoms containing granules, as determined by three independent two-sided *t*-tests (6 wt%: *h* = 0, *p* = 0.076, 9 wt%: *h* = 0, *p* = 0.43, and 12 wt%: *h* = 0, *p* = 0.064). All the phantoms were transported successfully, resulting in a reliability rate of 100%.

#### Motion Sequence

The highest transportation rate was determined to be associated with the 9 wt% homogeneous gelatine phantoms. Therefore, sub-experiment 2 was conducted with these phantoms. In Figure 6, the transportation rate is graphically displayed for the 1–5 and 2–4 motion sequences. Statistical analysis between the 1–5 and 2–4 motion sequence show that there was no significant difference in the transportation rates; 2.54 ± 0.89 versus 3.68 ± 2.14 mg/s (*h* = 0, *p* = 0.32), respectively. However, it should be noted that only 3 out of 7 phantoms were successfully transported with the 2–4 motion sequence, resulting in a reliability of only 43%. A transportation velocity of 1.49 ± 0.51 mm/s (transportation efficiency of 37.2%) and 1.61 ± 0.77 (transportation efficiency of 18.3%) was found for the 1–5 and 2–4 motion sequence, respectively. When comparing the achieved transportation velocity with the maximum achievable transportation velocity of the two motion sequences, it can be seen that more slip occurs in the 2–4 motion sequence, which substantiates the lower reliability of this motion sequence. Considering the relatively small amount of successfully transported tissue phantoms and higher slip from the 2–4 motion sequence and considering the fact that there was no statistically significant difference between the transportation rate of the 1–5 and 2–4 motion sequence, the choice was made to perform subsequent experiments with the 1–5 motion sequence.

#### *In vitro* Minced Meat Transportation Experiment

In an effort to test the prototype in a setting more closely mimicking the clinical situation, the ability, as well as the transportation rate and reliability, of the prototype to transport minced meat was evaluated using the 1–5 motion sequence and a rotational velocity of the electromotor of 46 rpm. For this experiment, the same experimental facility was used as described in Section “Experimental Facility.” The cuboid containers were filled with a 20 mm thick layer of minced meat, after which they were translated over a distance *d* of 30 mm over the distal end of the prototype, before turning the system on. From this experiment, it was substantiated that the prototype was able to reliably (100%) transport compacted minced meat using the 1–5 motion sequence. The average transportation rate was 2.19 ± 1.14 mg/s.

## Discussion

The ovipositor-inspired transportation mechanism described in this study was designed to overcome sub-optimal transportation behaviour modes that occur within aspiration-based transportation mechanisms. The principle at work in this prototype shows that a friction-based alternative to suction can be utilized within MIS instruments in order to reliably and successfully transport tissue through a slender tube. The potential of the proposed transportation mechanism lies in its miniaturizability and its independence of transportation length. The proposed method of transportation is not subject to the length dependent effects such as pressure loss, head loss and boundary layer growth that are associated with aspiration. Even so, the transportation rate of our prototype is still far below that of aspiration-based transport (Driessen et al., 2014). Clinically available morcellator reach transportation rates of in between 6.2 and 40.4 g/min. In comparison, the developed transportation system has a maximum transportation rate of 0.25 g/min, which is in between 50 and 162 times lower than clinically available morcellators. Even so, the developed transportation device can be beneficial over aspiration-based transportation devices when operating in environments surrounded by delicate structures, such as in the brain or spine, when transporting solids out of liquid environments, such as the bladder or coronary arteries, or when fast removal of tissue is not a major concern, such as in biopsies. The highest transportation rate of 0.25 g/min was achieved with the 9 wt% homogeneous gelatine tissue phantoms and the 1–5 motion sequence. The softer and stiffer gelatine tissue phantoms were transported with a significantly lower transportation rate. This finding indicates that there might be an optimal tissue stiffness range in which the prototype functions. It can be hypothesized that transportation of soft incoherent tissues might be challenging with the developed prototype, as soft tissue will potentially deform or shear near the blade surface; inhibiting transportation. For stiff, incompressible, tissues, on the other hand, the working principle might be hindered by the lack of tissue deformation. We, therefore, hypothesize that the transportation rate will follow a bell-shaped trend line with respect to tissue elasticity. No statistically difference in the transportation rate was found for the heterogeneous phantoms, indicating that heterogeneity of the phantoms might not affect tissue transport.

In order to increase the transportation rate of the prototype and minimize the dependence on tissue elasticity, multiple steps can be taken. The simplest solution to this problem is to increase the rotational velocity of the electromotor (by picking a different electromotor, for example). As of now, the electromotor is operating at a low rotational velocity of 46 rpm, this can easily be increased by removing the gear box from the electromotor. Furthermore, if microstructures are added to the inside of the tube of the prototype, the transportation rate may be increased. The inspiration for this is the wasp ovipositor. The inside of the ovipositor is covered with a microstructure, which prevents the egg from moving in any other direction than the desired transportation direction (Rahman et al., 1998; Matushkina, 2011). Similar structures can also prevent retrograde movement of the tissue within the lumen of our design. In unison with the addition of microstructures, different motion sequences could be researched. It was found that the 1–5 motion sequence provides a higher transportation rate and is more reliable than the 2–4 motion sequence. This is potentially due to the fact that the difference in friction force between retreating and advancing blades is higher in the 1–5 motion sequence than in the 2–4 motion sequence. In future, the number of blades could be increased to enlarge the friction differential between the advancing and retreating blades to improve reliability and the transportation rate. Furthermore, increasing the number of blades could also be beneficial when the tissue does not completely fill the lumen of the prototype or when we take the effect of gravity, and thus the orientation of the prototype, into account, as the chance of having a minimum of three blades into contact with the tissue increases. Another option to increase the transportation rate is to increase the stroke length or velocity with which the blades retreat and advance. Finally, the transportation mechanism can be combined with aspiration, potentially increasing the transportation rate and allowing for transporting incoherent tissues and liquids.

In order to incorporate the proposed prototype into a future medical instrument there are some key improvements, which warrant further investigation. First of all, the prototype design and manufacturing processes need to be optimized in the near future. The current design of the prototype did not include a radial locking mechanism such as the olistheter mechanism in the wasp’s ovipositor. The repeatability and uniformity of the parallel motion of the blades for extended periods of time will be increased if a method of radial locking is added to the blades. As of now, the electromotor is controlled by tuning the voltage, which does not allow for precise controlling and real-time readout of the rotational velocity during operation. In a future clinical prototype, a closed-loop controller could be implemented in the handle for precise control and monitoring of the rotational velocity of the electromotor during tissue transportation. Furthermore, from the conducted tests it can be concluded that the prototype is able to transport gelatine with varying densities and minced meat effectively. However, in order to assess the prototype’s functioning in a clinical setting, *ex-* or *in-vivo* tests should be conducted. These experiments will shed light on the effects of friction-based tissue transportation on the quality of the transported tissue sample and the effect of different material characteristics on the transportation rate and reliability. It is also worthwhile to test the prototype with a rotary cutting morcellator in combination with the transportation function to allow for tissue resection and transportation in one instrument. Finally, the testing of the transportation mechanism at different angles, especially vertically, may provide valuable information about the effects of gravity on the transportation process. Gravity can potentially have an effect on tissue transportation, by (1) increasing the normal force, and thus the friction force, between specific blades and the tissue in a situation where the shaft is between a horizontal and vertical alignment, or (2) by working against the friction force in a completely vertical alignment of the transportation mechanism. In the first situation, the friction differential between the retreating and advancing blades will be affected, whereas in the latter situation, this friction differential is unaffected, but the friction force between the blades and tissue needs to be sufficiently high to overcome gravity in order to initiate tissue transport. In an effort to minimize these effects, the number of blades could be increased or microstructures could be added.

By adding extra functionality to the prototype and further miniaturizing the outer dimensions, the potential application area of the transportation prototype can be significantly increased. By changing the blades into flexible cables, we are able to (1) make the device flexible for use in catheter interventions and (2) decrease the outer dimensions of the device. Cables are off-the-shelf available in a large variety of sizes, materials, and configurations, allowing for easy miniaturization and manufacturing of the prototype. Using cables as blades has been successfully demonstrated in the ultrathin self-propelling needles (∅0.4 mm) that rely on the same working principle for propelling themselves forward into tissue as the transportation mechanism uses for tissue transport (Scali et al., 2019). We are confident that with minor alterations, this needle can be converted into a tissue transportation mechanism. One of the main challenges to overcome when redesigning this system for tissue transportation is keeping the (flexible) blades in their position and preventing the lumen to collapse. This can be solved by using smart guidance structures around the cables. Furthermore, we can add steerability to the device by using the same bevel-tip steering mechanism used in the self-propelling needle (Scali et al., 2019) or we could use some of the cables for active steering by applying pulling forces. Lastly, we are investigating using fibre-optic cables as blades to allow for tissue sensing, adding a compliant griper at the tip for grasping tissues, and adding a cutting blade as the distal end to allow for simultaneous tissue transportation and resection.

The proposed prototype in this study has shown that friction-based transport has the potential of becoming a viable and reliable alternative to aspiration-based transportation. With the ongoing drive for performing MIS at remote locations in the body, the proposed prototype might fill the need for longer and thinner transportation instruments in future.

## Data Availability Statement

The raw data supporting the conclusions of this article will be made available by the authors, without undue reservation.

## Author Contributions

AS and IS: writing of the manuscript. PP, IS, AS, and PB: design of the prototype. IS: performing the experiment. MS and PB: critical revision of the manuscript. EK and PB: advice on structure and content of manuscript. All authors contributed to the article and approved the submitted version.

## Conflict of Interest

The authors declare that the research was conducted in the absence of any commercial or financial relationships that could be construed as a potential conflict of interest.

## Supplementary Material

Supplementary Video 1 Video illustrating the working principle of the developed wasp-inspired transportation device.
